# 20% Caloric Restriction did not Impact Bone Health nor Exercise-Induced Elevations in Bone Mass in Young Female Rats

**DOI:** 10.1007/s00223-025-01469-4

**Published:** 2026-01-17

**Authors:** Ken D. Sumida, Brady Slater, Haley Folta, Kassandra Lee, Sarina Karwande, Quinton Wong, S. Victoria Jaque, Frank Frisch

**Affiliations:** 1 Departments of Health Sciences, Chapman University, One University Drive, Orange, CA 92866 USA; 2 Department of Kinesiology, California State University, Northridge, CA USA

**Keywords:** Caloric restriction, Tibia, DXA, BMD, Bone mechanical properties

## Abstract

The purpose of this study was to determine if a 6-week 20% caloric restrictive (CR) diet, with or without resistance training, would impact bone health in growing young female rats. Forty female rats (~ 5 wks old) were randomly divided into the following groups: baseline (*n* = 8), sedentary fed a normal diet (N, *n* = 8), sedentary fed a 20% CR diet (D, *n* = 8), resistance trained fed a normal diet (NT, *n* = 8), and resistance trained fed a 20% CR diet (DT, *n* = 8). The exercise groups were conditioned to climb a vertical ladder 4 consecutive times (per exercise session) with weights appended to their tail 3 days/wk for a total of 6 wks. Tibial bone mineral density (BMD) was assessed via dual-energy x-ray absorptiometry scans. After 6 wks, the body mass (mean ± SD) for CR fed groups (D = 232.6 ± 26.3 g & DT = 216.6 ± 17.9 g) were significantly lower than the normal fed groups (*N* = 266.1 ± 31.5 g & NT = 251.9 ± 23.4 g). Tibial BMD (in g/cm^2^) for the sedentary CR group (D = 0.184 ± 0.005) was not significantly different compared to the sedentary normal fed group (*N* = 0.184 ± 0.010). Resistance training resulted in an elevation in BMD (NT = 0.195 ± 0.011 & DT = 0.192 ± 0.004) compared to the sedentary groups). The results indicate that during the growth period in young female rats, a 20% CR diet did not impact tibia BMD, nor did CR alter the resistance training-induced elevation in BMD.

## Introduction

Caloric restrictive diets alone or combined with exercise have typically been utilized to reverse obesity [1]. However, with the advent of social media, there is a growing trend to lose body mass via diet, or a combination of diet and exercise, even by normal weight young women to become thin attributable to its association with health and/or beauty [2]. In support, Wharton et al. [3] reported over 73% of young American women attempted to lose weight even though 78% were normal or underweight. Similarly, Latimer et al. [4] reported that 28% of American college students were trying to lose weight even though they were under- or at a normal body weight. While employing diets in normal weight young females can reduce body weight, it can also lower bone mass [5]. In support, we previously observed lower bone mineral densities in the femur from non-obese women who reportedly consumed 55% of their recommended minimum daily requirement [6]. Further, a report from the CALERIE (Comprehensive Assessment of the Long-Term Effects of Reducing Intake of Energy) trails in healthy, non-obese men and women reported bone loss at osteoporotic fracture sites when they engaged in ~ 25% caloric restrictive diets [7]. In addition, young female athletes such as wrestlers, dancers, distance runners, figure skaters, and gymnasts (among others), strive to maintain a low body mass index (BMI) via caloric restriction to optimize athletic performance and/or achieve a specific weight class/category [8]. While exercise has the potential to elevate bone mass that can counter bone loss due to caloric restrictive diets [7], the amount of caloric restriction that can be implemented as well as the use of exercise during caloric restriction to counter any bone loss is unclear. This is due to the challenge in controlling for the various confounding variables in humans including (but not limited to) genetics, delayed menarche, amount of caloric restriction, type of exercise, duration of exercise, and intensity of training. Given the elevated risk for osteoporosis in women, understanding how to maintain bone health in normal weight young females who engage in diets, with or without exercise, is essential.

Animal studies can help to elucidate the impact of exercise in maintaining bone health during caloric restrictive diets by controlling many of the confounding variables associated with human studies. However, the animal studies investigating the impact of exercise during caloric restriction in females are inconsistent. Some have reported a positive effect on bone following a forced treadmill running protocol 3–4 times per week in female rats during a 12-week, 40% caloric restrictive diet [9–11]. In contrast, others have reported a negative impact on bone employing a 30% caloric restrictive diet and voluntary daily wheel running in female mice [12] and young male rats [13]. The varying animal results may be related to the difference between forced treadmill running compared to daily wheel running where animals will engage in significantly more voluntary exercise.

Compared to treadmill running in animals, resistance training has the potential to provide a greater stimulus for bone formation [14, 15]. Our prior studies have demonstrated the use of ladder climbing as an effective stimulus that mimics resistance training resulting in an elevation in BMD in rats [16–23]. Supporting the caloric restriction treadmill studies [9–11], we previously reported this mode of exercise mitigated tibial bone loss during a 6-week, 40% caloric restrictive diet in young female rats [24]. While there is general concern that significant caloric restrictive diets in combination with endurance training can result in moderate to substantial bone loss in female animals [25, 26], the impact of a less severe caloric restrictive diet and the effects of resistance training on bone health remains to be determined. If normal weight young females engage in caloric restrictive diets for the purpose of athletics or aesthetics, then the amount of caloric restriction and the impact of resistance training during caloric restriction to maintain bone health would contribute to minimizing the severity or reducing the risk of osteoporosis.

Therefore, the purpose of the study was to determine the impact of moderate caloric restriction (i.e., 20%) with and without a resistance training program on BMD in growing, young female rats. We also measured serum markers of bone turnover and bone mechanical properties via 3-point bending tests. We hypothesized that moderate caloric restriction would not impact bone health, nor would it impact the resistance training-induced elevation in BMD and bone mechanical properties.

## Methods

### Animals

The experimental protocol for this study was pre-approved by the Chapman University Institutional Review Board and in accord with all national guidelines for the care and use of animals for research. Forty female Sprague Dawley rats (initially ~ 140 g, ~ 5 weeks old), obtained from Charles River Laboratories (Wilmington, MA), were housed individually and maintained on a reverse l2/12 hour, light/dark cycle. The animals were acclimated to their living conditions for 1 week after which eight animals were randomly sacrificed for baseline data (BL). The remaining 32 animals were randomly separated into the following groups: a sedentary normal diet fed group (N, *n* = 8), a sedentary caloric restrictive diet fed group (D, *n* = 8), a resistance trained normal fed diet group (NT, *n* = 8), and a resistance trained caloric restrictive diet fed group (DT, *n* = 8).

### Caloric Restriction

Based upon body weight, animals were pair-fed where a normal *ad libitum* sedentary fed animal (N) was matched with a corresponding sedentary diet restricted animal (D). In like manner, a normal *ad libitum* fed resistance trained animal (NT) was matched with a corresponding resistance trained caloric restricted animal (DT). The amount of food consumed by the normal *ad libitum* fed animal was determined every day at the same time of day (8 a.m.). The matched caloric restriction animal was then given 80% of the food eaten by the normal *ad libitum* fed animal the preceding day resulting in a 20% caloric restrictive diet. All normal *ad libitum* fed animals (N and NT) were allowed free access to food from a normal diet (Product No. D12450B) provided by Research Diets, Inc. (New Brunswick, NJ). Caloric restrictive diet groups (D and DT) were fed a modified diet (Product No. D14060201) provided by Research Diets, Inc. that was supplemented with additional vitamins and minerals so that the only variable was 20% fewer calories via a reduction in carbohydrates (Table 1). All animals had free access to water.

**Table 1:** Diets

Normal diet (D12450B)		Caloric restriction (D14060201)	
Protein, Casein	200.00 g	Protein, Casein	200.00 g
Protein, Cystine	3.00 g	Protein, Cystine	3.00 g
Carbohydrate, Sucrose	350.00 g	Carbohydrate, Sucrose	248.00 g
Carbohydrate, Starch	315.00 g	Carbohydrate, Starch	208.00 g
Carbohydrate, Maltodextrin	35.00 g	Carbohydrate, Maltodextrin	35.00 g
Cellulose, BW200	50.00 g	Cellulose, BW 200	50.00 g
Fat, Soybean oil	25.00 g	Fat, Soybean oil	25.00 g
Fat, Lard	20.00 g	Fat, Lard	20.00 g
Dicalcium phosphate	13.00 g	Dicalcium phosphate	13.00 g
Calcium carbonate	5.50 g	Calcium carbonate	5.50 g
Potassium citrate	16.50 g	Potassium citrate	16.50 g
Choline bitartrate	2.00 g	Choline bitartrate	2.00 g
Mineral mix (see below)	10.00 g	Mineral mix (see below)	10.00 g
Vitamin mix (see below)	10.00 g	Vitamin mix (see below)	10.00 g
Total	1055.00 g	Total	846.00 g

Mineral mix	
Sodium chloride	51.80 g
Magnesium sulfate	51.52 g
Magnesium oxide	8.38 g
Ferric citrate	4.20 g
Manganese carbonate hydrate	2.45 g
Zine carbonate	1.12 g
Chromium potassium sulfate	0.39 g
Copper carbonate	0.21 g
Ammonium molybdate tetrahydrate	0.06 g
Sodium fluoride	0.04 g
Sodium selenite	0.01 g
Potassium Iodate	0.01 g
Sucrose	179.21 g

Vitamin mix	
Vitamin E Acetate	10.00 g
Niacin	3.00 g
Biotin	2.00 g
Pantothenic acid	1.60 g
Vitamin D3	1.00 g
Vitamin B12	1.00 g
Vitamin A	0.80 g
Pyridoxine	0.70 g
Riboflavin	0.60 g
Thiamine	0.60 g
Folic acid	0.20 g
Menadione sodium bisulfite	0.08 g
Sucrose	978.42 g

*Diets do not contain any soy protein or phytoestrogens

### Resistance Training

The strength training regimen has previously been described [16–24]. Briefly, the resistance trained animals were required to climb a 1-meter vertical ladder 4 consecutive times with weights (i.e., fishing sinkers) appended to their tail. The exercised animals trained 3 days/week for a total of 6 weeks. Since the NT and DT animals were handled on training days, the N and D sedentary animals were also handled on the same days and times to equalize any stress attributable to human contact. All animals were weighed at the beginning of the week to monitor weight gain and, for the resistance trained animals, to determine the amount of weight to append to their tails for the remainder of the week. The resistance training protocol involved the following weekly progression. Animals started with 30% body mass (BM) appended to their tail and each week the amount of weight was elevated by 30% BM until week 5 where they carried 135% of their BM. At week 6 they were carrying 150% of their BM per exercise session.

### Experimental Protocol

To minimize any residual effect of the last training bout, all animals were sacrificed 48 h after the final 6-week exercise session along with all sedentary animals. The Flexor Hallucis Longus (FHL) was rapidly dissected from the right hindlimb, weighed, and immediately frozen in liquid nitrogen for the subsequent determination of protein content. The left hindlimb was rapidly amputated, positioned, and frozen in liquid nitrogen for the assessment of BMD of the tibia. As previously described by Turner and Burr [27], the tibia from the right hindlimb was dissected and placed in an ethanol/saline (50/50) solution until the subsequent measurement of bone mechanical properties (via three-point bending tests). Blood samples were collected, allowed to clot, centrifuged, and the serum was frozen for the subsequent measurement of serum osteocalcin (OC) and pyridinoline (PYD). All tissue and serum samples were kept at -80 °C until its analyses.

### Chemical Analyses

Protein concentration in the FHL was assessed [28] as an indirect indicator of a training adaptation as initially reported by Hornberger and Farrar [29]. A sandwich enzyme-linked immunosorbent assay (ELISA, Biomedical Technologies, Inc., Stoughton, MA) was used to determine serum osteocalcin levels (an indicator of osteoblast activity). The intra-assay and inter-assay variation was < 2.5%. Serum pyridinoline (an indicator of osteoclast activity) was measured using a competitive enzyme immunoassay (EIA, Quidel Corp., San Diego, CA). The intra- and inter-assay variation was < 5%. A microplate reader (MaxLine, Molecular Devices Corp., Sunnyvale, CA) was used with the absorbance set at 450 nm for the ELISA and 405 nm for the EIA. A standard curve was generated for all chemical analyses and controls were run to ensure quality. For all standard curves, the Pearson’s Product Correlation Coefficient for linear relationships (i.e., protein), or the Coefficient of Determination for non-linear curves, (i.e., OC and PYD) was greater than 0.98.

### Bone Mineral Density

A Dual-Energy X-ray Absorptiometer (DXA - GE Lunar Prodigy, Chicago, IL) employing the small animal software module (version 6.81) was used to assess the BMD of the entire left tibia. Briefly, the left hindlimb was thawed, positioned, and the entire limb was scanned. Condyle and malleolus curvatures of the tibia were used as anatomical markers to ensure proper positioning and to prevent twisting so that the curvatures were not exaggerated or obliterated. Three consecutive measurements were performed with the hindlimb repositioned between each scan. The reported BMD was the average of three scans and the coefficient of variation (calculated as [mean/SD]x100) for repeated scans (mean ± standard error) was 1.50 ± 0.69%.

### Three-Point Bending Tests

Bone mechanical properties were measured within one week of dissection using a three-point bending rig via a texture analyzer instrument (TA-XT2, Texture Technologies, Ramona, CA) that is identical in function and data acquisition to an Instron. Prior to testing, the right tibial bone was removed from the ethanol/saline solution and submerged in saline for 24 h prior to testing at room temperature. Following calibration procedures, the tibia was patted dry and secured to the rig located on the stage of the texture analyzer. The span of the two support points was 14 mm and the deformation rate was 0.9 mm/sec. A medial to lateral force was applied to the midshaft of the bone. The maximal load to failure (Fmax, units = Newtons), energy to failure (EF, determined from the area under the load-deformation curve to the fracture point, units = Newtons x mm), and Stiffness (determined from the initial slope, units = Newtons/mm) were assessed using Texture Expert (v. 1.22, Stable Micro Systems Ltd., Surrey, England, UK).

### Calculations & Statistics

A Power Analysis based upon an expected BMD from our prior study employing a 40% CR diet [24] was used to determine the sample size for each group. Based upon our prior study, the expected BMD difference between means from normal fed and CR fed groups was 0.010 and the expected SD was 0.006. To achieve a power of 80% and a level of significance of 5%, a sample size of 8 per group was determined. Next, work (i.e., training volume, expressed in Joules) was calculated as the product of the total mass lifted by the animal (body weight plus the amount of weight appended to the tail), the acceleration due to gravity, and the distance covered. Due to the differences in body mass between NT and DT during the 6-week diet, the amount of work (i.e., training) was divided by the body weight of the animal and the results expressed as Joules/100 g body weight. A Student’s t-test was used to determine statistical significance with respect to training volume between NT vs. DT. Total protein in the FHL was calculated as the product of protein concentration and muscle mass. A one-way ANOVA was used to compare BL to all other groups. A two-factor (Diet x Exercise) ANOVA was employed for all comparisons (except for BL). A Tukey’s post hoc test used if a significant F ratio was identified. The level of significance set was at *P* < 0.05 for all statistical comparisons and the results were expressed as the mean ± standard deviation (SD).

## Results

**Fig. 1:**
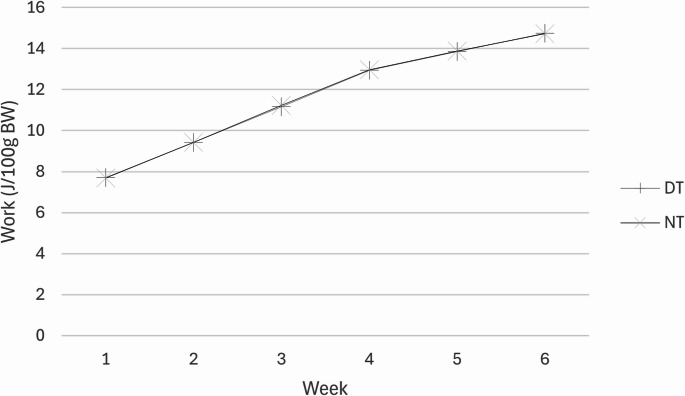
Work (Joules/100 grams of body weight) performed each week for DT (Resistance trained group fed a 20% Caloric restricted diet, *n* = 8) and NT (Resistance trained group fed a normal diet, *n* = 8). Data are expressed as the mean ± SD. SD bars are depicted, but too small to visualize. No significant difference between groups

The initial body weight was not significantly different between groups (Table 2). After the 6-week experimental period, the significant increase in body weight observed for *ad libitum* fed groups compared to BL supports the typical growth pattern for female rats. There were no interaction effects, however, there was a main effect of diet. Specifically, following the 6-week 20% caloric restrictive diet, the final body weight was significantly lower for caloric restricted fed groups compared to normal *ad libitum* fed groups (Table 2). Next, the relative training volume was not significantly different between NT and DT groups (Fig. 1). As before, there were no interaction effects, but in support of a resistance training outcome, there was a significant main effect on the FHL mass and protein content (Table 2) that was significantly higher for resistance trained groups (NT & DT) compared to the sedentary groups (N & D).

**Table 2:** Body mass and Flexor Hallucis Longus

Group	Initial BM (grams)	Final BM (grams)	FHL Mass (grams)	FHL protein (mg protein/muscle)
BL	136.7 ± 8.9		0.084 ± 0.012†	14.2 ± 0.9†
N	139.2 ± 9.9	266.1 ± 31.5	0.122 ± 0.017	21.6 ± 2.5
D	139.9 ± 7.4	232.6 ± 26.3§	0.128 ± 0.016	21.7 ± 2.5
NT	137.3 ± 7.9	251.9 ± 23.4	0.144 ± 0.014*	25.9 ± 3.1*
DT	136.1 ± 7.1	216.6 ± 17.9§	0.130 ± 0.014*	22.8 ± 1.1*

BM = Body Mass, FHL = Flexor Hallucis Longus. BL = Baseline group (n= 8), N = Sedentary normal fed group (n=8), D = Sedentary 20% Caloric restricted fed group (n= 8), NT = Normal fed resistance trained group (n=8), DT = 20% Caloric restricted fed resistance trained group (n=8). Data are expressed as the mean ± SD. The BL and FHL data was compared to all groups via ANOVA. †Significant difference between BL vs. all other groups. With the exception of BL, data for all other groups were analyzed via a 2-way ANOVA (Diet x Exercise). There were no interaction effects (*P* > 0.05). §Significant main effect of diet on body mass, 20% Caloric restriction (D and DT) vs. Normal Fed (N and NT). *Significant main effect of resistance training (NT and DT) on FHL Mass (*P*=0.039) and FHL protein (*P*=0.037) vs. Sedentary (N and D)

**Fig. 2:**
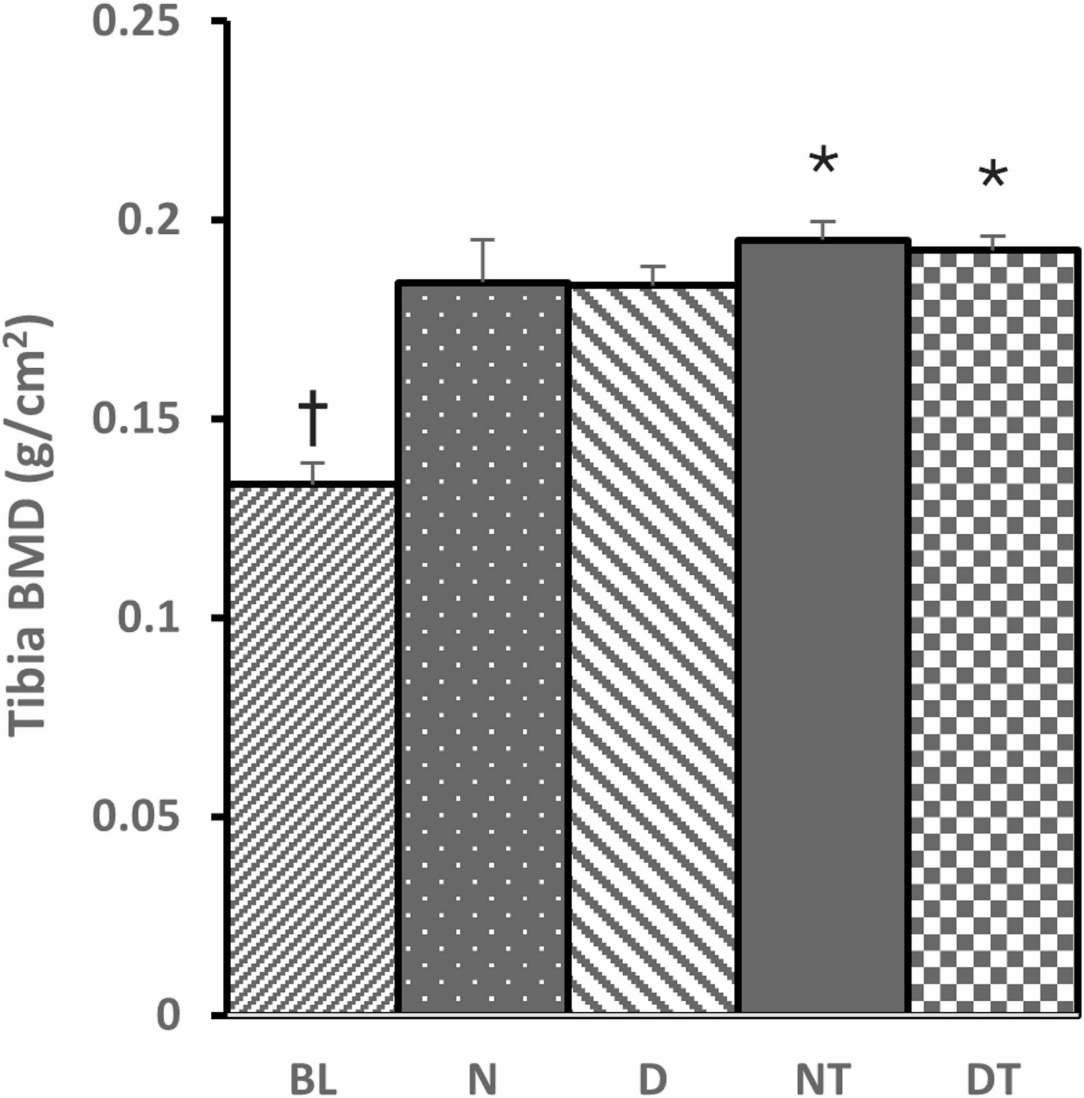
BMD = Bone Mineral density (g/cm^2^) for the tibia. BL = Baseline group (*n* = 8), N = Sedentary Normal Fed Group (*n* = 8), D = Sedentary 20% Caloric Restricted Fed Group (*n* = 8), NT = Normal Fed Resistance Trained Group (*n* = 8), and DT = 20% Caloric Restricted Fed Resistance Trained Group (*n* = 8). Data are expressed as the mean ± SD. The BL data was compared to all groups via ANOVA. **†**Significant difference between BL vs. all other groups. With the exception of BL, data for all other groups were analyzed via a 2-way ANOVA (Diet x Exercise). There were no interaction effects (*p* = 0.74) nor main effect of diet (*p* = 0.56). *Significant main effect of exercise (*p* < 0.05), i.e., Resistance Training (NT and DT) compared to Sedentary (N and D)

**Fig. 3:**
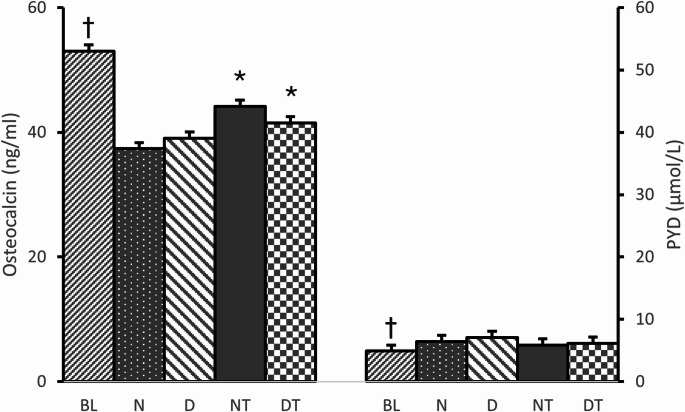
Serum biomarkers: Osteocalcin (ng/ml) and PYD = Pyridinoline (µmol/L). BL = Baseline group (*n* = 8), N = Sedentary Normal Fed Group (*n* = 8), D = Sedentary 20% Caloric Restricted Fed Group (*n* = 8), NT = Normal Fed Resistance Trained Group (*n* = 8), and DT = 20% Caloric Restricted Fed Resistance Trained Group (*n* = 8). Data are expressed as the mean ± SD. The BL data was compared to all groups via ANOVA. **†**Significant difference between BL vs. all other groups. With the exception of BL, data for all other groups were analyzed via a 2-way ANOVA (Diet x Exercise). There were no interaction effects for OC (*p* = 0.34) or PYD (*p* = 0.59) nor main effect of diet for OC (*p* = 0.82) or PYD (*p* = 0.20). *Significant main effect of exercise (*p* < 0.05), i.e., Resistance Training (NT and DT) compared to Sedentary (N and D)

There were no interaction effects and no main effect of diet on tibial BMD between normal *ad libitum* fed groups (N & NT) and the pair-matched caloric restriction fed groups (D & DT). However, there was a main effect of exercise where resistance trained groups (NT & DT) had significantly greater tibial BMD compared to the sedentary groups (N & D). Specifically, resistance training resulted in a 5.4% increase in BMD (Fig. 2). In support, there were no interaction effects on serum OC, but there was a main effect of exercise on serum OC that was significantly higher for resistance trained groups compared to the sedentary groups. There were no interaction nor main effects on serum PYD (Fig. 3).

**Fig. 4:**
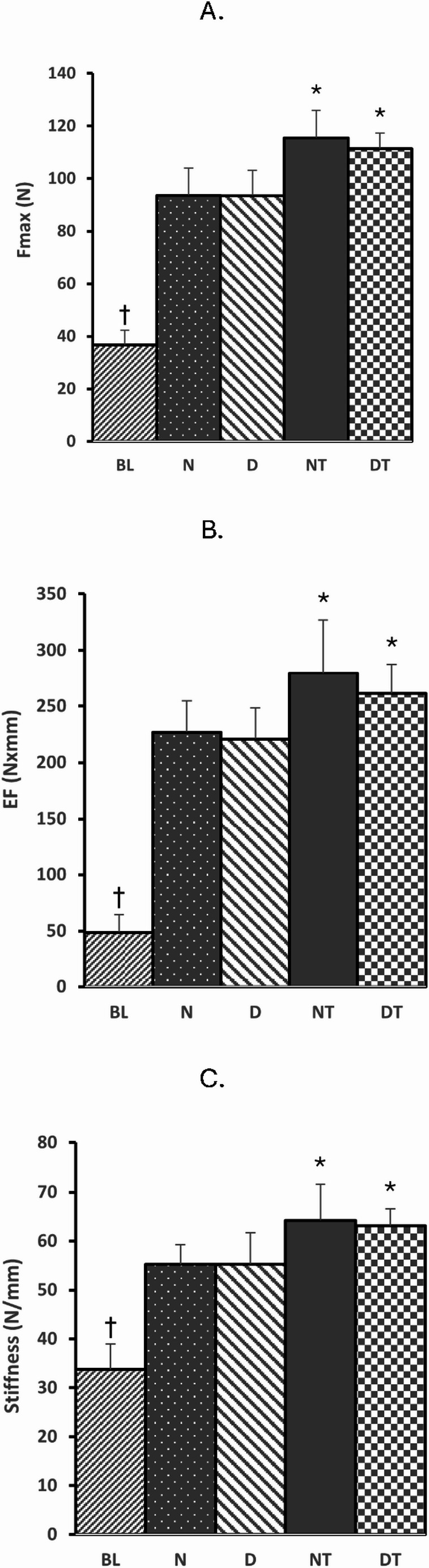
Bone mechanical properties.** A** Fmax = amount of force to break/fracture the bone (N = Newtons). **B** EF = Energy to Failure determined from the area under the load-deformation curve to the fracture point (Newtons x mm).** C** Stiffness = determined from the initial slope of the load-deformation curve (Newtons/mm). BL = Baseline group (*n* = 8), N = Sedentary Normal Fed Group (*n* = 8), D = Sedentary 20% Caloric Restricted Fed Group (*n* = 8), NT = Normal Fed Resistance Trained Group (*n* = 8), and DT = 20% Caloric Restricted Fed Resistance Trained Group (*n* = 8). Data are expressed as the mean ± SD. The BL data was compared to all groups via ANOVA. **†**Significant difference between BL vs. all other groups. With the exception of BL, data for all other groups were analyzed via a 2-way ANOVA (Diet x Exercise). (**A**) There were no interaction effects (*p* = 0.56) nor main effect of diet (*p* = 0.53). (**B**) There were no interaction effects (*p* = 0.60) nor main effect of diet (*p* = 0.30). (**C**) There were no interaction effects (*p* = 0.77) nor main effect of diet (*p* = 0.80). *Significant main effect of exercise (*P* < 0.05), i.e., Resistance Training (NT and DT) compared to Sedentary (N and D)

There were no interaction effects and no main effect of diet on bone mechanical properties (Fig. 4). However, there was a main effect of exercise that supports the training-induced elevations in BMD. The Fmax (Fig. 4A), EF (Fig. 4B), and Stiffness (Fig. 4C) were significantly greater for resistance trained groups (NT & DT) compared to the sedentary groups (N & D). Resistance training resulted in a 21.3% increase in Fmax, a 20.8% increase in EF, and a 15.3% increase in Stiffness.

## Discussion

The 20% reduction in calories resulted in no interaction effects and no main effects of diet on BMD and bone mechanical properties between the caloric restricted fed groups (D & DT) compared to normal *ad libitum* fed groups (N & NT). In addition, the caloric restrictive diet did not alter the training-induced elevation in tibial BMD or bone mechanical properties as supported by the significant main effect of resistance training for both DT and NT compared to their sedentary counterparts. Therefore, the results support our hypothesis that moderate caloric restriction (i.e., 20%) did not compromise bone health, nor did it impact the resistance training-induced elevation in BMD and bone mechanical properties in young growing female animals.

Incorporating exercise during weight loss regimens in overweight and obese individuals has been appropriately advocated to help maintain bone integrity [30]. However, the impact of exercise on bone health in young female athletes who engage in exercise and are not overweight are inconsistent. Burckhardt et al. [31] observed the tendency for lower bone mineral content in adolescent ballet dancers who engaged in diets to maintain a low BMI. In like manner, low BMDs have been reported in high school female distance runners with low BMI [32]. In contrast, reports in elite adolescent female figure skaters [33] and college gymnasts [34] with low BMI tended to have greater BMD. Determinants such as age, menarche, nutrient consumption (among others) can contribute to the differences between these young female athletic populations. It may also be a combination of the amount of caloric restriction, the length of time caloric restriction is employed, the type of exercise, training intensity, frequency, and/or exercise duration that can compromise, maintain, or enhance bone integrity.

Despite the ability to control confounding variables, animal studies have similarly produced conflicting results when combining exercise and caloric restriction. Implementing a 12-week, 40% energy restrictive diet, and incorporating treadmill running has been reported to offer a partial protection from tibial bone loss [9, 10] as well as the femur [11] in female rats. In agreement with these treadmill studies, we administered a 40% caloric restrictive diet for 6 weeks in growing female rats where our resistance training protocol similarly mitigated tibial bone loss [24]. However, several studies reported a deleterious effect of exercise training upon the bone in combination with caloric restriction. McGrath et al. [12] implemented a 30% caloric restrictive diet and voluntary wheel running in female mice and reported that 6 weeks of chronic exercise resulted in more tibial bone loss than caloric restriction alone. Similarly, Aikawa et al. [35] employed a 12-week 30% caloric restricted diet in combination with voluntary wheel running in young female rats and observed greater declines in BMD of the tibia. Hattori et al. [13] examined the impact of voluntary wheel running combined with a 30% caloric restrictive diet for 13 weeks in young male rats and also reported greater declines in tibial BMD and bone mechanical properties in caloric restricted exercise trained animals. The discrepancy in these prior studies compared to the current report and treadmill studies may be related to the elevated amount of voluntary wheel running that contributes toward greater reductions in energy availability in addition to the caloric restrictive diet. Compared to the BL group, our implementation of a 20% caloric restrictive diet suggests sufficient energy intake to maintain skeletal muscle growth and bone health in young growing female animals. Further, compared to voluntary wheel running, our resistance training protocol suggests less exercise energy expenditure to complete the work that did not disrupt the training-induced elevations in FHL, BMD, and bone mechanical properties.

Collectively, there may be a threshold amount of caloric restriction that does not compromise bone health. The potential for a threshold caloric restriction limit whereby bone integrity can be maintained is supported by Aikawa et al. [36] who examined different amounts of caloric restrictive diets in young female rats where essential nutrient intake was equivalent compared to normal fed animals except for lower energy availability in the form of carbohydrates. After 10.5 weeks, they reported no change in bone integrity compared to normal fed animals when implementing a 10% and 20% caloric restrictive diet whereas a 30% and 40% caloric restriction lowered BMD in the tibia, femur, and lumbar spine [36]. The current study supports the findings by Aikawa et al. [36] where a 20% diet preserves tibial bone integrity if essential vitamins and minerals are maintained and calories are the only deficit in consumption. In a prior study, we found no difference in the BMD of the femur from women who consumed 80% of the recommended minimum daily allowance (RMDA) of calories compared to women who consumed 100% of their RMDA where both groups reported exercising for about an hour every other day [6]. Based on a 3-day nutritional history, there were no significant differences between the 80% RMDA and 100% RMDA groups regarding protein, calcium, phosphorus, and Vitamin D intake [6]. Taken together, bone integrity can be maintained despite the decline in caloric intake of 10% and 20% as reported in the animal study by Aikawa et al. [36], our current report, and our prior human study [6]. However, we recognize the potential for any amount of caloric restriction (that is supplemented with essential nutrients) whereby bone health is maintained will require further investigations in humans given other potential factors such as the length of time a caloric restrictive diet is employed, age, sex differences, race, and the use of tobacco and/or alcohol (to name a few).

The maintenance of bone health during moderate weight loss and the benefits of strength training have been examined in separate reviews of human studies by Hunter et al. [37] and a more recent review by Liu and Rosen [38]. The current results in animals support the benefits of resistance training as well as quantify the potential amount of caloric restriction that will not impact the training-induced elevation in BMD. In addition, the current report confirms previous findings in humans [39–41] and animals [17, 18, 20–24, 42] pertaining to the exercise-induced elevation in osteoblast activity as the potential mechanism for the elevation in BMD as supported by the higher serum OC for the resistance trained groups. Our results regarding training-induced elevations in bone mechanical properties of the tibia are also consistent with our prior reports [16–19, 22, 24] and others that have examined the femur after jumping exercise [14] and tower climbing [15] in rats. It also supports prior studies whereby small elevations in BMD can result in large increases in bone mechanical properties [14–19, 22, 24].

Finally, we recognize several limitations of this study. First, the DXA measures mass per area where differences in bone size can result in misinterpretations with the use of DXA [43]. Next, we acknowledge that having access to micro-CT (micro-computed tomography) or pQCT (peripheral quantitative computed tomography) would have added to our interpretation of the data pertaining to bone architecture and structural parameters. Further, the measurement of various hormones such as insulin-like growth factor, parathyroid, leptin and/or estrogen would have added to the interpretation of our results. Despite all these limitations, the bone mechanical properties support the training-induced elevations in BMD.

In summary, given the absence of a cure for osteoporosis, prevention is of utmost importance. Engaging in significant caloric restrictive diets in normal weight, non-athlete young females for the purpose of being thin is unwarranted since it can compromise bone health. Further, bone integrity can be jeopardized in young normal weight female athletes who engage in significant diets to maintain a low BMI in order to participate in a lower weight class or to optimize performance. However, if normal weight young females engage in diet and exercise, then understanding the amount of caloric restriction in combination with the type of exercise that can maintain bone health is essential to help minimize the onset or reduce the severity of osteoporosis later in life. Therefore, to the extent the current results in young female animals can be extrapolated to humans, if diets are employed by normal weight young women, moderate caloric restriction (i.e., 20%) can be implemented without compromising bone health if a decline in energy intake is the only variable. In addition, moderate caloric restriction in combination with resistance exercise does not impact the training-induced elevation in bone mass and bone mechanical properties.
